# Next-generation sequencing-based genetic landscape and its clinical implications for Chinese acute myeloid leukemia patients

**DOI:** 10.1186/s12935-018-0716-7

**Published:** 2018-12-22

**Authors:** Xin-xin Cao, Hao Cai, Yue-ying Mao, Qi Wu, Lu Zhang, Dao-bin Zhou, Jian Li

**Affiliations:** Department of Hematology, Peking Union Medical College Hospital, Chinese Academy of Medical Sciences & Peking Union Medical College, 1 Shuai Fu Yuan Hu Tong, Dongcheng District, Beijing, 100730 People’s Republic of China

**Keywords:** Acute myeloid leukemia, Induction chemotherapy, Next-generation sequencing, Older patients

## Abstract

**Background:**

Acute myeloid leukemia (AML) is a clinically and biologically heterogeneous disease. The survival of older patients is generally poor. In the current study, we sought to investigate the differences in molecular gene mutations between younger and older AML patients, and to identify those newly diagnosed AML patients who are more likely to respond to standard cytarabine and daunorubicin induction chemotherapy.

**Methods:**

We retrospectively evaluated 179 patients who were newly diagnosed with non-M3 AML. A next-generation sequencing assay covering 34 genes was used to investigate recurrently mutated genes. The mutational status of fusion genes was determined by real time PCR.

**Results:**

The median age at diagnosis was 53 years (range 18–88 years). Sixty-eight patients were 60 years or older with a median age of 67 years (range 60–88 years). Eighteen patients (10.1%) carried t(8;21)(q22;q22.1) or *RUNX1*–*RUNX1T1* gene fusion, and there was a significantly higher incidence in younger patients (p = 0.019). At least one non-synonymous gene mutation was detected in 159 patients (88.8%). The median number of gene mutations was two (range 0–6). The mean number of molecular gene mutations at diagnosis was higher in older patients than younger patients (2.5 vs 1.83, p = 0.003). Older patients had significantly higher incidences of *ASXL1* (22.1% vs 13.5%, p = 0.025) and *TP53* mutations (13.2% vs 3.6%, p = 0.034). In total, 78 patients received DA60 (daunorubicin 60 mg/m^2^ per day on days 1–3 and cytarabine 100 mg/m^2^ twice per day on days 1–7) as the induction therapy, and information was available on their response to induction treatment. Patients with *RUNX1*–*RUNX1T1* gene fusion were significantly more likely to achieve complete remission (CR) after DA60 induction therapy (p = 0.026), as were patients without the *ASXL1* mutation (p = 0.007).

**Conclusion:**

Older AML patients had a lower incidence of favorable cytogenetics and higher frequencies and burdens of molecular mutations that are associated with poor prognosis compared to younger patients. Patients with *RUNX1*–*RUNX1T1* gene fusion or without the *ASXL1* gene mutation had a better chance of achieving CR when treated with cytarabine and daunorubicin induction chemotherapy.

## Background

Acute myeloid leukemia (AML) is a clinically and biologically heterogeneous disease characterized by the clonal expansion of undifferentiated myeloid precursors. Although induction chemotherapy with cytarabine and daunorubicin has been shown over the past 40 years to improve clinical outcomes in younger patients, survival for older patients remains very poor [1–3]. In addition to patient factors, such as old age, poor performance status and concomitant comorbidity, genetic factors are also related to outcomes in older AML patients [4–6].

The AML genome is one of the simplest cancer genomes, which makes it amenable to the clinical use of targeted next-generation sequencing (NGS). Recently, a combination of cytogenetic analysis and mutation testing has been integrated into the classification and risk assessment of AML patients [7–9]. The European LeukemiaNet revised the prognostic model for AML in 2017 to add mutations in *RUNX1* and *ASXL1* to the previously identified molecular risk categories defined by mutations in *NPM1*, *CEBPA*, *FLT3*–*ITD* and *TP53*. This model stratifies AML patients into three prognostic groups (good, intermediate and poor risk). However, recent advances in the understanding of the molecular alterations in AML are mostly derived from younger patients [9]. Some studies have demonstrated that the chromosome abnormalities and gene mutation patterns are different among older AML patients [7, 8, 10]. Less is known about the differences in gene mutations between younger and older patients with AML, especially Chinese patients.

The aim of this study was to comprehensively investigate the differences in clinicobiological features and molecular gene mutations between younger and older AML patients, and to identify those newly diagnosed AML patients who are more likely to respond to standard cytarabine and daunorubicin induction chemotherapy.

## Methods

### Patients

All 179 adult patients who were newly diagnosed with non-M3 AML according to the FAB criteria [11] at Peking Union Medical College Hospital (Peking, China) between July 2015 and April 2018, and for whom complete clinical, cytogenetic and laboratory data before treatment were available, were included in this study. Bone marrow samples from all patients underwent mutational analysis by NGS. Informed consent was obtained from all patients and the protocol was approved by the Peking Union Medical College Hospital Ethics Committee. The present study was performed in accordance with the ethical standards of the 1964 Declaration of Helsinki and its later amendments.

### Cytogenetics and fusion genes analysis

The bone marrow samples were investigated using G-banding analysis and karyotyped according to the International System for Human Cytogenetic Nomenclature.

The mutational status of fusion genes such as *RUNX1*–*RUNX1T1 and CBFβ*–*MYH11* was determined by real time PCR (RT-PCR). In addition, RT-PCR was used to detect 11 MLL-related common fusion genes, including MLL-AF1q, MLL-AF1p, MLL-AF4, MLL-AF9, MLL-AF10, MLL-AF6, MLL-ELL, MLL-ENL, MLL-AFX, MLL-SEPT6 and MLL-AF17. We used Multiplex RT-PCR Fusion Gene Kits provided by Rightongene.

### Next-generation sequencing

Analyses were conducted of the relevant mutations of 34 genes, including *ASXL1, BCOR, BCORL1, CALR, CBL, CEBPA, CSF3R, DNMT3A, ETV6, EZH2, FLT3, GATA2, IDH1, IDH2, JAK2, KIT, KMT2A, KRAS, MPL, NPM1, NRAS, PDGFRA, PHF6, PIGA, RUNX1, SETBP1, SF3B1, SH2B3, SRSF2, TET2, TP53, U2AF1, WT1* and *ZRSR2.*

Read pairs were aligned to Refseq hg19 (downloaded from the UCSC Genome Browser, URLs) by the Burrows–Wheeler Aligner version 0.7.13-r1126. Samtools version 1.3 was used to generate chromosomal coordinate-sorted BAM files. We used targeted next-generation sequencing with the Rightongene AML/MDS/MPN Sequencing Panel (Rightongene). The NGS libraries were paired-end sequenced (2 × 150 bp) on an Illumina MiSeq System (Illumina, San Diego, CA). The mean depth of each sample was 2500×, with an average 5% of the target sequence being covered sufficiently deeply for variant calling. Samtools mpileup was applied for SNV/indel calling and filter workflow.

Gene mutations were assigned to functional groups similar to those previously described as follows [12, 13]: DNA methylation and hydroxymethylation-related—*DNMT3A*, *TET2* and *IDH1/2*; RNA spliceosome—*SF3B1*, *SRSF2, ZRSR2* and *U2AF1*; chromatin remodeling—*ASXL1*, *EZH2, BCOR* and *KMT2A*; transcriptional deregulation—*CEBPA*, *RUNX1* and *WT1*; activated signaling—*NRAS*, *KRAS, CBL, KIT, JAK2* and *FLT3*.

### Statistical analysis

Complete remission (CR) was defined according to the criteria of the International Working Group [14]. The discrete variables of patients with and without specific molecular alteration were compared using the Fisher exact test. The Fisher exact test was used to compare categorical variables, whereas the Mann–Whitney test was used to compare continuous variables between groups. We performed all statistical analyses using SPSS version 21 software (IBM Corp., Armonk, NY, USA), and considered p-values of less than 0.05 to be statistically significant.

## Results

### Clinical characteristics

Of 179 newly diagnosed AML patients, 116 were males and 63 were females (Table 1). The median age at diagnosis was 53 years (range 18–88 years). Sixty-eight patients were 60 years or older with a median age of 67 years (range 60–88 years). Five patients were diagnosed as treatment-related AML (one was older patient), and 21 patients were diagnosed as secondary AML (ten with myelodysplastic syndrome, three with chronic myeloid leukemia, three with chronic myelomonocytic leukemia, two with myelofibrosis, two with essential thrombocythaemia and one with myeloproliferative neoplasm). Among the 21 secondary AML patients, nine were older patients. There were no differences in gender, white blood cells (WBC) or blasts in the bone marrow between younger patients and older patients.

**Table 1:** Clinical manifestations and cytogenetic abnormalities of AML patients stratified by age

	Total (n = 179)	Younger patients (n = 111)	Older patients (n = 68)	p value
Age, years (median, range)	53 (18–88)	44 (18–59)	67 (60–88)	
Male (n, %)	116 (64.8)	71 (64.0)	45 (66.2)	0.872
WBC (× 10^9^/L) (median, range)	7.82 (0.4–362.6)	8.48 (0.4–362.6)	7.42 (0.6–271.4)	0.380
Blast cells in BM (%, range)	53 (10–95)	57 (10–94.5)	53 (21–95)	0.824
Complex karyotype	16/130 (12.3)	9/81 (11.1)	7/49 (14.3)	0.784
*RUNX1*–*RUNX1T1*	18 (10.1)	16 (14.4)	2 (2.9)	0.019
*CBFβ*–*MYH11*	10 (5.6)	8 (7.2)	2 (2.9)	0.322

*WBC* white blood cell, *BM* bone marrow

### Molecular gene mutations of patients

Chromosome data were available for 130 patients at diagnosis, including 63 patients (48.5%) with a normal karyotype and 16 patients (12.3%) with a complex karyotype. Eighteen patients (10.1%) carried t(8;21)(q22;q22.1) or *RUNX1*–*RUNX1T1* gene fusion, while ten patients (5.6%) carried inv(16)(p13.1q22) or t(16;16)(p13.1;q22) or *CBFβ*–*MYH11* gene fusion. Younger patients had a significantly higher incidence of t(8;21)(q22;q22.1) or *RUNX1*–*RUNX1T1* gene fusion (p = 0.019) (Table 1). We also found two patients had *MLL*-*AF9*, one patient had *MLL*-*ELL*, one patient had *MLL*-*ENL* and one patient had *MLL*-*SEPT6* gene fusion.

**Fig. 1:**
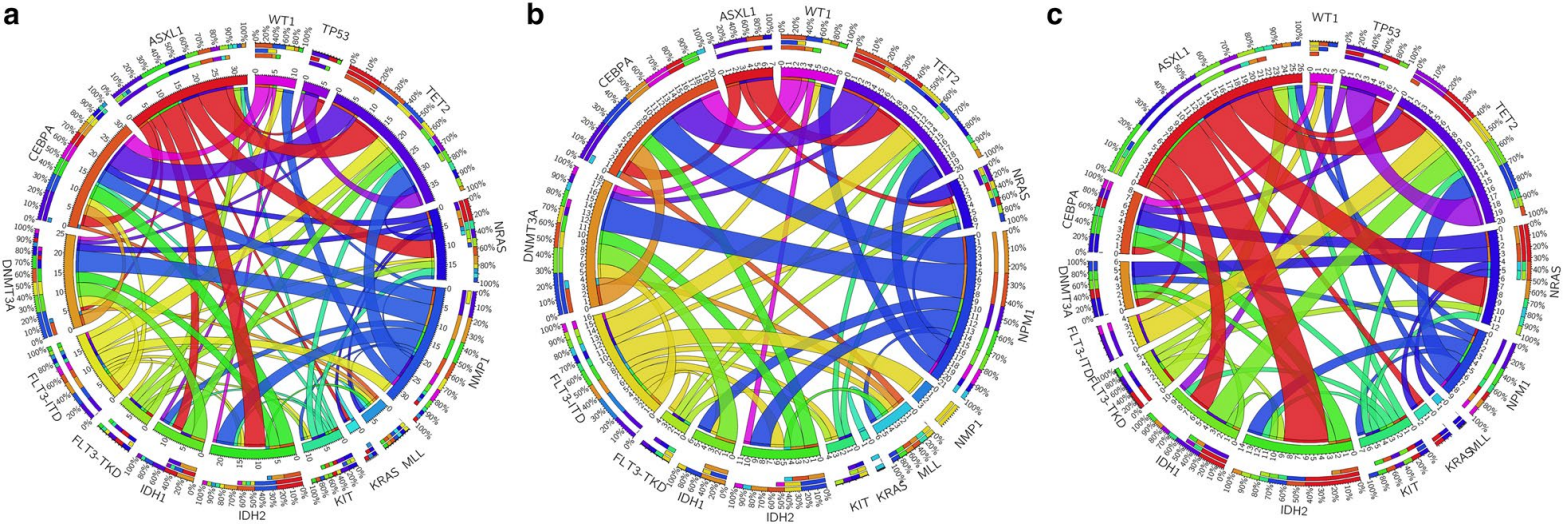
Circos plots depicting the relative frequencies and pairwise co-occurrences of selected common genetic alterations: in all AML patients (**a**), separately in patients 60 years or older (**b**) and in patients younger than 60 years (**c**). The length of the arc corresponds to the frequency of the first gene mutation, and the width of the ribbon corresponds to the proportion of co-occurrence with the second gene mutation. Both the distribution of gene mutations and the pattern of mutation co-occurrences appear to be distinct between older and younger AML patients

At least one non-synonymous gene mutation was detected in 159 patients (88.8%). The median number of gene mutations was two (range 0–6). The distributions of molecular gene mutations are shown in Fig. 1. The most common molecular event in the total cohort was a *CEBPA* mutation (17.9%), followed by *TET2* (16.8%), *ASXL1* (14.0%), *NRAS* (11.7%), *NPM1* (11.2%), *IDH2* (10.1%) and *FLT3*–*ITD* mutations (10.1%) (Table 2). Both the distribution of gene mutations and the pattern of mutation co-occurrence appear to be distinct between older and younger AML patients (Fig. 1). The mean number of molecular gene mutations at diagnosis was higher in older patients than younger patients (2.5 vs 1.83, p = 0.003). Older patients had significantly higher incidences of *ASXL1* (22.1% vs 13.5%, p = 0.025) and *TP53* (13.2% vs 3.6%, p = 0.034) mutations. The older patient group also showed a trend of more *DNMT3A* (14.7% vs 5.4%, p = 0.056) and *RUNX1* (11.8% vs 4.5%, p = 0.081) mutations than the younger patient group. Moreover, younger patients showed a trend of more *KIT* (10.8% vs 2.5%, p = 0.083) and *NRAS* (15.3% vs 5.9%, p = 0.092) mutations. Among the different gene functional groups, the older patient group had a significantly higher incidence of DNA methylation- and hydroxymethylation-related genes (48.5% vs 27.0%, p = 0.004), RNA spliceosome (30.9% vs 14.4%, p = 0.013) and chromatin remodeling (29.4% vs 10.8%, p = 0.002) gene mutations, while the younger patient group had a significantly higher incidence of activated signaling gene mutations (37.8% vs 19.1%, p = 0.012).

**Table 2:** Association of age with molecular genetic alterations

	Total (n = 179)	Younger patients (n = 111)	Older patients (n = 68)	p value
Mutation number (mean)	2.08	1.83	2.50	0.003
*CEBPA*	32 (17.9)	22 (19.8)	10 (14.7)	0.428
*TET2*	30 (16.8)	15 (13.5)	15 (22.1)	0.153
*ASXL1*	25 (14.0)	10 (9.0)	15 (22.1)	0.025
*NRAS*	21 (11.7)	17 (15.3)	4 (5.9)	0.092
*NPM1*	20 (11.2)	12 (10.8)	8 (11.8)	1
*IDH2*	18 (10.1)	10 (9.0)	8 (11.8)	0.768
*FLT3*–*ITD*	18 (10.1)	12 (10.8)	6 (8.8)	0.800
*DNMT3A*	16 (8.9)	6 (5.4)	10 (14.7)	0.056
*KIT*	14 (7.8)	12 (10.8)	2 (2.9)	0.083
*TP53*	13 (7.3)	4 (3.6)	9 (13.2)	0.034
*RUNX1*	13 (7.3)	5 (4.5)	8 (11.8)	0.081
*IDH1*	12 (6.7)	5 (4.5)	7 (10.3)	0.216
*WT1*	9 (5.0)	7 (6.3)	2 (2.9)	0.486
*GATA2*	5 (2.8)	2 (1.8)	3 (4.4)	0.370
*FLT3*–*TKD*	5 (2.8)	3 (2.7)	2 (2.9)	1
*KRAS*	4 (2.2)	3 (2.7)	1 (1.5)	1
DNA methylation	63 (35.2)	30 (27.0)	33 (48.5)	0.004
RNA splicing	37 (20.7)	16 (14.4)	21 (30.9)	0.013
Chromatin architecture	32 (17.9)	12 (10.8)	20 (29.4)	0.002
Transcriptional deregulation	50 (27.9)	31 (27.9)	19 (27.9)	1
Activated signaling	55 (30.7)	42 (37.8)	13 (19.1)	0.012

Patients with t(8;21)(q22;q22.1) or *RUNX1*–*RUNX1T1* gene fusion had a median one gene mutation (range 0–3). Seven (38.9%) also had a *KIT* mutation (p < 0.001), and none had a *CEBPA* mutation (p = 0.046).

*CEBPA* mutations were detected in 32 patients, and 11 of those patients (6.1%) were bi-allelic. Five patients with a *CEBPA* mutation also had a *GATA2* mutation, while none of the *CEBPA* wild-type patients had a *GATA2* mutation (p < 0.001) (Table 3). Among the 25 patients who had an *ASXL1* mutation, eight patients also had a *TET2* mutation (p = 0.041), six also had an *IDH2* mutation (p = 0.023) and four had a SETBP1 mutation (p = 0.008). Among the 20 subjects with an *NPM1* mutation, six also had a *DNMT3A* mutation (p = 0.004) and five also had an *IDH2* mutation (p = 0.034). We also noted that *IDH2* and *TET2* mutations were mutually exclusive. None of the 30 subjects who had a *TET2* mutation had an *IDH2* mutation (p = 0.046). None of the other co-mutated genes reached statistically significant incidence.

**Table 3:** Correlations among different mutations

	*CEBPA* ^Mut^	*CEBPA* ^WT^	p value
*GATA2* ^Mut^	5	0	
*GATA2* ^WT^	27	147	< 0.001

	*ASXL1* ^Mut^	*ASXL1* ^WT^	p value
*TET2* ^Mut^	8	22	
*TET2* ^WT^	17	132	0.041
*IDH2* ^mut^	6	12	
*IDH2* ^WT^	19	142	0.023
*SETBP1* ^mut^	4	3	
*SETBP`* ^WT^	21	151	0.008

	*NPM1* ^Mut^	*NPM1* ^WT^	p value
*DNMT3A* ^Mut^	6	10	
*DNMT3A* ^WT^	14	149	0.004
*IDH2* ^mut^	5	13	
*IDH2* ^WT^	15	146	0.034

	*TET2* ^Mut^	*TET2* ^WT^	p value
*IDH2* ^Mut^	0	18	
*IDH2* ^WT^	30	131	0.046

*Mut* mutant, *WT* wild type

### Correlations between mutations and clinical outcomes

Thirty-three patients went back to their local hospital for further chemotherapy after diagnosis in our center. Eight patients only received best support therapy. In total, 138 patients received induction chemotherapy. Among them, 78 patients received DA60 (daunorubicin 60 mg/m^2^ per day on days 1–3 and cytarabine 100 mg/m^2^ twice per day on days 1–7) as the induction therapy and information was available on their response to induction treatment. Fifteen of those patients were 60 years or older, and the overall median age was 46 years (range 18–73 years). Forty-five patients (57.7%) achieved a CR following one course of induction therapy. We found patients with *RUNX1*–*RUNX1T1* gene fusion were significantly more likely to achieve a CR after DA60 induction therapy (p = 0.026) as well as patients without the *ASXL1* mutation (p = 0.007).

## Discussion

Genetic mutations in AML patients that escape cytogenetic detection are increasingly being discovered, and these mutations may serve as potential markers to extend the prognostic parameters in AML. An understanding of the frequencies of gene mutations in AML patients can facilitate the selection of targeted therapies and help us understand the potential pathways or resistance mechanisms. In the current study, we found 88.8% of AML patients had at least one mutation detected by targeted NGS. This figure is higher than what has previously been reported in the literature [15], which may be due to the larger gene panel we used. This high incidence of gene mutation detection further justifies the performance of NGS on AML patients as it can yield, at high frequency, genetic information that may be clinically actionable.

*CEBPA* was found to have the highest mutation rate in our cohort, which was higher than that reported in the literature [16, 17] but similar to previous reports on Chinese AML patients [18, 19]. We detected *NPM1* and *FLT3* mutations at frequencies of 11.2% and 12.9%, respectively, which is similar to the results reported by Hussaini et al. [20]. However, other groups have reported frequencies ranging from 20 to 33% [5–10, 19, 21]. This difference may be due to the different patient populations, but we cannot exclude technical differences as different laboratories use different mutation detection techniques and algorithms for calling mutations.

In our study we confirmed that patients with *RUNX1*–*RUNX1T1* gene fusion had a significantly higher incidence of *KIT* mutation. We also found some gene mutations were correlated (e.g. *CEBPA* and *GATA2*; *ASXL1* and *TET2*, *IDH2*, *SETBP1*; *NPM1* and *DNMT3A*, *IDH2*). On the other hand, some gene mutations appeared to be potentially mutually exclusive (e.g. *TET2* and *IDH2*). However, the clinical and biological significances of these correlations or exclusions of gene mutations are unknown.

Although much effort has been made to clarify the correlation between molecular changes and clinical outcomes of AML patients, most of the data pertain to younger patients. In our study, we consecutively recruited newly diagnosed AML patients for whom sufficient samples were available for mutation analysis without restriction of age, so we could compare the genetic alterations between younger and older AML patients. First, we showed that younger patients had more *RUNX1*–*RUNX1T1* gene fusion than older patients. Second, older patients had more gene mutations than younger patients. Furthermore, older patients had higher incidences of *ASXL1*, *TP53*, DNA methylation- and hydroxymethylation-related, RNA spliceosome and chromatin remodeling gene mutations, and also exhibited a trend of higher incidences of *DNMT3A* and *RUNX1* mutations. Younger patients had higher incidences of activated signaling gene mutations. Several genetic alterations were found to have prognostic significance and have been incorporated into risk stratification of AML [8]. Previously, *RUNX1*–*RUNX1T1* gene fusion was considered a good prognostic factor and *TP53* and *ASXL1* mutations were poor prognostic factors [8, 22]. Our study confirmed *TP53* and *ASXL1* mutations are prevalent in older patients. Taken together, the data suggest the pathogenesis of AML among younger patients vs older patients may be different. In addition to a lower incidence of favorable cytogenetics, the higher frequencies and burdens of molecular mutations that are associated with poor prognosis in older patients might explain the dismal outcome in this patient group.

The use of targeted NGS testing is useful for the identification of the population of patients who have an excellent chance of achieving a CR when treated with cytarabine and daunorubicin induction chemotherapy. We have shown that patients who have a *RUNX1*–*RUNX1T1* gene fusion or do not have an *ASXL1* gene mutation constitute a group whose chance of achieving a CR is higher. An alternative induction therapy or use of a novel agent may need to be considered in patients with *ASXL1* gene mutation.

## Conclusion

In conclusion, our data indicate clinically targeted NGS sequencing frequently detects gene mutations in AML patients (more than 85%). Older AML patients had a lower incidence of favorable cytogenetics and higher frequencies and burdens of molecular mutations that are associated with poor prognosis than younger patients. Patients with a *RUNX1*–*RUNX1T1* gene fusion or without an *ASXL1* gene mutation have a better chance of achieving a CR when treated with cytarabine and daunorubicin induction chemotherapy.

## Abbreviations

AML: acute myeloid leukemia
CR: complete remission
DA60: daunorubicin 60 mg/m^2^ per day on days 1–3 and cytarabine 100 mg/m^2^ twice per day on days 1–7
NGS: next-generation sequencing
RT-PCR: real time PCR
