# Correction to: Next-generation sequencing-based genetic landscape and its clinical implications for Chinese acute myeloid leukemia patients

**DOI:** 10.1186/s12935-019-0736-y

**Published:** 2019-01-14

**Authors:** Xin-xin Cao, Hao Cai, Yue-ying Mao, Qi Wu, Lu Zhang, Dao-bin Zhou, Jian Li

**Affiliations:** Department of Hematology, Peking Union Medical College Hospital, Chinese Academy of Medical Sciences & Peking Union Medical College, 1 Shuai Fu Yuan Hu Tong, Dongcheng District, Beijing, 100730 People’s Republic of China

## Correction to: Cancer Cell Int (2018) 18:215 10.1186/s12935-018-0716-7

Following publication of the original article [1], we have been notified that the data in Fig. 1 was wrongly presented. The correct Fig. 1 is presented below.

**Fig. 1:**
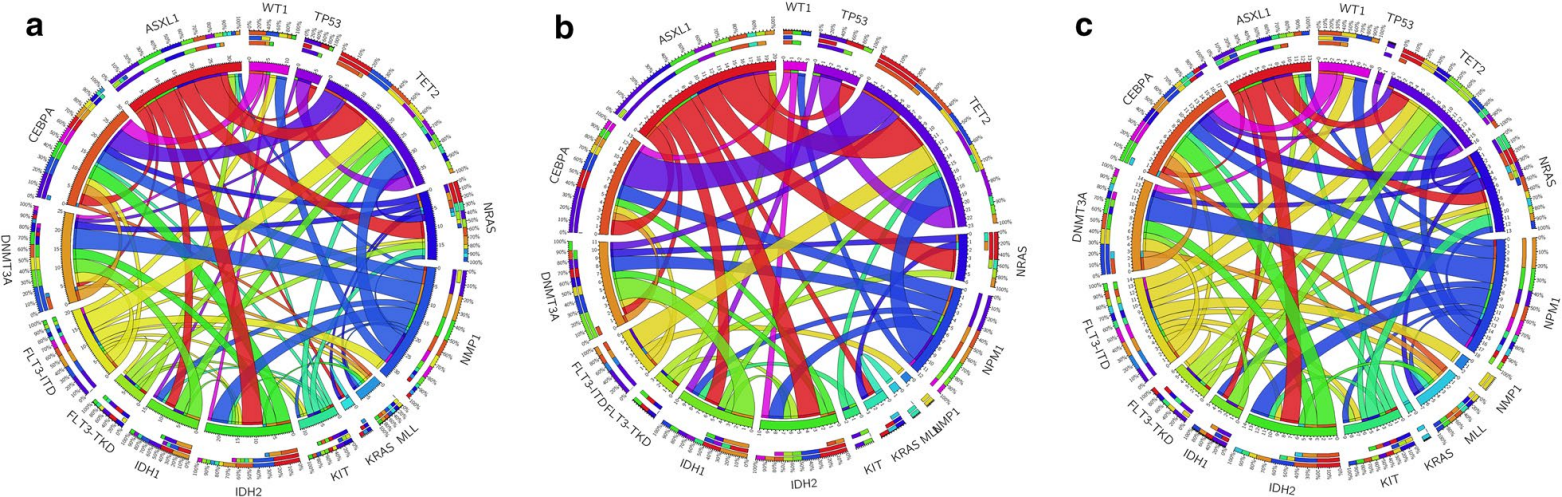
Circos plots depicting the relative frequencies and pairwise co-occurrences of selected common genetic alterations: in all AML patients (**a**), separately in patients 60 years or older (**b**) and in patients younger than 60 years (**c**). The length of the arc corresponds to the frequency of the first gene mutation, and the width of the ribbon corresponds to the proportion of co-occurrence with the second gene mutation. Both the distribution of gene mutations and the pattern of mutation co-occurrences appear to be distinct between older and younger AML patients
